# Vitality and wound-age estimation in forensic pathology: review and future prospects

**DOI:** 10.1080/20961790.2018.1445441

**Published:** 2018-03-29

**Authors:** Na Li, Qiuxiang Du, Rufeng Bai, Junhong Sun

**Affiliations:** a Department of Forensic Pathology, Shanxi Medical University, Taiyuan, China;; b Key Laboratory of Forensic Science, Shanxi Medical University, Taiyuan, China;; c Key Laboratory of Evidence Science, China University of Political Science and Law, Beijing, China;; d Collaborative Innovation Centre of Judicial Civilization, Beijing, China

**Keywords:** Forensic sciences, forensic pathology, wound age, vitality, estimation

## Abstract

Determining the age of a wound is challenging in forensic pathology, but it can contribute to the reconstruction of crime scenes and lead to arrest of suspects. Forensic scholars have tended to focus on evaluating wound vitality and determining the time elapsed since the wound was sustained. Recent progress in forensic techniques, particularly high-throughput analyses, has enabled evaluation of materials at the cellular and molecular levels, as well as simultaneous assessment of multiple markers. This paper provides an update on wound-age estimation in forensic pathology, summarizes the recent literature, and considers useful additional information provided by each marker. Finally, the future prospects for estimating wound age in forensic practise are discussed with the hope of providing something useful for further study.

## Introduction

Determining the age of a wound is challenging in forensic pathology, but it can contribute to the reconstruction of crime scenes and lead to the arrest of a suspect [1–3]. Forensic pathologists must identify the timing and order of injuries in cases involving multiple traumas by different offenders because punishment typically varies according to the severity of the injury. In cases of violent death, the main focus is on (1) whether an injury was caused while the individual was alive or during the agonal or postmortem period, and (2) how long the victim survived after the wound was inflicted [4].

After infliction of a wound, a series of vital reactions (e.g. haemorrhage, inflammatory cell infiltration, formation of granulation tissue) must be taken into consideration to obtain convincing proof of antemortem injury. These antemortem reactions are collectively termed “vitality”, which is related to whether the victim was alive at the time of the trauma and how long before the death of the victim the trauma was inflicted [5]. Wound vitality can be evaluated using morphological, cytological, and molecular biological techniques. A number of biomarkers involved in vital reactions reportedly increase the accuracy of wound-age estimation [6,7].

It is widely believed, however, that there are no established parameters or methods that yield these data because of the non-specificity, poor repeatability, and inadequate diagnostic performance of biomarkers and the limitations of the techniques used. Therefore, systematic and specific criteria for identifying useful markers are needed, and more advanced techniques should be applied to generate data with enhanced accuracy and objectivity. Because wound-age estimation is an intricate and multifactorial problem — similar to weather forecasting — use of a combination of several parameters could reduce the errors in wound-age estimation.

Another obstacle is the availability of, and the not negligible ethical issues involved with, using human specimens with a known time of death [7–9]. Therefore, animal studies are essential, but the applicability of the results to humans lacks definitive supporting evidence. Thus, issues regarding how to transfer the results obtained in animal models to humans and how to utilize effectively the large amount of data generated to determine the timing of injury remain unresolved [5,7].

Considering that Chinese forensic scholars have made great progress in improving the estimation of wound age in recent years, articles from the China National Knowledge Infrastructure (CNKI) database (the most influential database in China) deserve attention. Hence, studies on wound-age estimation were systematically searched the PubMed and CNKI with a primary search strategy, i.e. [(wound-age estimation) OR (wound-age determination) OR (wound-age evaluation) OR (time course of wound) OR (timing of wound) OR (wound aging)] AND [(forensic medicine) OR (legal medicine) OR (medical jurisprudence)]. These searches — which were filtered by containing the full text, English language, and publication date to 12 December 2016 on the first search — yielded 643 articles in total from PubMed. Among them, 337 articles appeared during the period 20102016, which accounted for more than 50% of all of the PubMed articles. In CNKI, 188 articles were found to the date 31 December 2016, with 64 articles appearing since 2010, which accounted for more than one-third of all the CNKI articles. In addition, Kondo et al. [10] had conducted a review of the molecular pathology of wound healing and summarized the articles before 2010. Thus, based on these two important points, the resulting matches from 2010 to 2016 were screened and reviewed. The useful additional information gained by evaluating the valuable markers was analysed, and the future prospects for wound-age estimation in forensic practise addressed, hoping to provide useful data for further study.

## Common tissues used to date wounds

Because of the frequency at which they are encountered in forensic practise, the skin, skeletal muscle, and brain tissue around inflicted wounds were the most examined tissues in reviewed studies, with the trauma having involved an incision or contusion. For example, because brain damage is frequently involved in cases of violent death and is typically fatal, much research has focused on estimating wound age in cases involving brain damage [11–13]. Skeletal muscle and skin have also been the subjects of most of the recent experimental and investigative studies, respectively.

The articles we reviewed were principally experimental and investigative studies. Generally, in experimental studies, the time after wounding at which samples are taken is predetermined, whereas in investigative studies a number of specimens are collected at various time points after the wounding. The studies in our review involved mainly animal and autopsy specimens, although some samples were from living human subjects.

Animal experiments have the advantage of being controllable, which increases the reproducibility and reliability of the results. They also facilitate investigation of the process of wound repair, which is a basic physiological response and is similar in human and animals. The time after injury can also be controlled. The extent to which the results are applicable to humans, however, remains unclear [7,14], which suggests that markers with a high level of sequence homology should be used because they are likely to display similar functions during wound repair. It is also possible to use human samples as a calibration standard for animal data to improve the accuracy of injury time estimations in forensic practise.

Autopsy specimens are the most accurate and realistic samples, particularly if the wound age is known. However, the availability of these samples is limited because of missing information and insufficient documentation. Additionally, even when samples of wounds with a known age are collected, the time at which the wounding occurred can vary widely and, in some cases, it was not determined during the period of interest. Moreover, postmortem changes (e.g. putrefaction, decomposition, desiccation), the individual’s age, wound location, survival time, and clinical history (to mention only some of the factors) must be considered. In these circumstances, controlled *ex vivo* putrefaction should be applied [1]. Additionally, strict control of the collection criteria increases the reliability of the results.

Samples from living human subjects are obtained mainly from patients with skin disease and diseases that require surgical resection, as well as patients referred to a forensic physician. These samples have accurate time records and are typically preserved up to the time of interest. Thus, they offer two distinct advantages: their human origin and the accuracy of their time data. Such samples are usually not from healthy persons, however, and their *in vitro* preservation suppresses vital reactions. At times, wound age must be determined in living subjects because macroscopic assessment of an injury is inadequate for medicolegal purposes. Ethical issues related to the use of tissue from living donors are also important. Indeed, the use of humans in research is subject to approval by the local ethics committee [8,9]. Moreover, as samples from injuries sustained by live human subjects must be obtained in a non-invasive manner (swabbing), so the sample size is frequently inadequate, leading to unreliable, sometimes false-negative results [8,9,15].

## Methods for wound-age estimation *Morphological analysis*

Various methods are used to determine wound age. Morphological analysis has a long history of being the most commonly used method because of its visual nature or intuitionistic nature, objectivity, and ability to evaluate marker localization. Visual observation (colour changes) of the bruises has also long been an investigative tool to determine the age of a bruise [16]. It provides much useful information about wound aging and is still irreplaceable. Attempts have been made to determine the age of bruises based on their coloration seen by visual inspection, but this method has been shown to be too variable to be of practical use because the time of appearance and the colour of a bruise are affected by the depth, location, and skin complexion, among other factors [17]. Thus, the problem must be approached using scientific experimental investigations, so we focus here on molecular techniques.

The potential use of numerous temporal markers in forensic pathology has therefore been explored. Although conventional histological evaluation (e.g. with haematoxylin-eosin and Berlin blue staining) can detect changes 6 h after an injury [18–20], its practical application is limited. Immunohistochemistry and immunofluorescence studies, which are useful for estimating the age of early-stage wounds, allow (1) localization of tissue factors indicative of the stage of response and (2) determination of the phases of activation of individual cells [6]. These data enable evaluation of the linkage of morphology with function and close in on the interval of the wound age determined by assays of cytokines and adhesion molecules. An immunofluorescence multiple-staining technique enables detection of three or four markers simultaneously and facilitates qualitative and quantitative analyses of tissue. As the percentages of polymorphonuclear neutrophils, mononuclear cells, and fibroblastic cells in injured zones reportedly change over time [18,20–22], they may have potential use in estimating wound age. Some studies have reported a positive correlation between an early wound stage and markers’ levels — even as early as 1998 when Dressler et al. [23] observed strongly positive immunohistochemical reactions for P-selectin 3 min after wound creation. The above findings will be useful in future studies. Notably, the distance inflammatory cells migrate from the free vessel — which has not been studied owing to the limitations of the measurement techniques — is theoretically related to an early stage of injury.

The results of quantitative immunohistochemical assays are reportedly not accurate or stable and may be influenced by operator skills, such as the subjective definition of positive standards. Such issues restrict the clinical application of immunohistochemistry [24]. Digital slice-scanning systems automatically eliminate subjective factors by identifying and examining different areas of a sample, and they facilitate investigations of the distance between inflammatory cells and the free vessel. The combination of a digital slice-scanning system and immunofluorescence multiple-staining techniques enables automated testing of three or four markers simultaneously and thus should be investigated further.

### Molecular biological analysis

Generally, during the first minutes or hours after wound infliction, histological analysis cannot determine whether a wound was sustained before or after death [14]. After wounding, however, the mRNA levels of cytokines and enzymes typically change sooner than the protein levels and the histomorphology [25–28]. Hence, assays based on mRNA are suitable for estimating the age of early-stage wounds. Although RNA is less stable than protein, it has been detected in a long-preserved sample [22]. Total RNA of sufficient quality and quantity can be obtained using biological stains that are several months, even years, old [29–31]. Thus, the mRNA levels of inflammatory cytokines and wound-healing factors are assayed using the real-time polymerase chain reaction (PCR) to evaluate wound age. Because real-time PCR (qPCR) is a highly sensitive method to detect even slight changes in gene expression among samples, it is imperative to be careful at every step, including data analysis [32–34]. Data normalization by employing reference genes is a critical step for accurate analysis to detect inevitable experimental variations, especially disparities in the amount of sample loading. It is an issue that the expression of some housekeeping genes is upregulated after injury [35,36], and it is important to identify a stably-expressed housekeeping gene after injury for effective normalization.

Currently, high-throughput methods (e.g. gene chip analysis, high-throughput sequencing, real-time PCR, 384 Microplate system) enable analysis of dozens to hundreds of genes simultaneously, which not only considerably reduces the cost of testing but also yields results with high repeatability and stability. These methods, which enable identification of markers used for estimating wound age, will likely be used to detect the differential expression of mRNAs after injury and will play an important role in future investigations.

Wound vitality and protein levels can also be evaluated using Western blotting and enzyme-linked immunosorbent assays, which are more sensitive than immunohistochemistry. Moreover, in contrast to genomics and transcriptomics, proteomics can provide insight into the signal transduction events that directly affect the biochemical processes of life. Comparisons of results between laboratories, however, are hampered by the complexity of the procedures and the difficulty of controlling conditions. Protein microarray is a sensitive high-throughput method that enables simultaneous analysis of multiple protein analytes in a single sample [37].

### Other methods

Mao et al. [4] used electric impedance spectroscopy to develop a new, rapid tool for estimating wound age. Zhang et al. [38] employed an isobaric tag for relative and absolute quantifications in conjunction with liquid chromatography-mass spectrometry/mass spectrometry to identify differentially expressed proteins as reliable biomarkers of diffuse axonal injury. These methods are not yet used frequently in legal medicine but do show promise for the future.

Each method has advantages and disadvantages. Red blood cell extravasation, which is examined by conventional histology, is considered a sign of vital reactions. Because it can also appear postmortem, however, it is not a reliable marker of wound vitality [1,14]. Therefore, morphological and molecular parameters should be used in combination to reduce the error when determining the time at which a wound was inflicted [6,24]. High-throughput analysis, whether at the mRNA or protein level, is a critical methodological advance.

### Biomarkers of wound aging

#### Skin and skeletal muscle injury

Wound healing is a complex process that occurs in response to tissue injury, including skin and muscle tissue. Wound healing comprises inflammatory, proliferative, and maturation phases, which involve interactions between various cell types and soluble factors [25,39–41]. During the inflammatory phase, a variety of chemo-kines are released at the injured site, leading to the recruitment of inflammatory cells, such as neutrophils and macrophages. At the proliferation stage, in skin, re-epithelialization and newly formed granulation tissue begin to cover the wound area to complete tissue repair. In skeletal muscle, satellite cells, a population of postnatal muscle stem cells, begin to proliferate and undergo differentiation into myocytes. They then fuse with either each other or damaged myofibres to repair injured muscle and fibrotic tissue [42–44].

Infiltration by inflammatory cells is an indicator of tissue repair [1,6,41,45]. Forensic pathologists, unlike general pathologists, tend to focus on chronologically mapping the appearance and disappearance of inflammatory cells or substances secreted during the inflammatory process. These phenomena — e.g. the proportion of positive cells, level of tissue fibrosis, and distance between inflammatory cells and the free vessel — are influenced by the degree of injury, which affects the accuracy of wound-age determination. Therefore, it is necessary to establish models with different degrees of injury and assess the parameters involved in wound healing to determine the precise time of the injury.

The levels of mRNA and proteins involved in tissue repair (e.g. adhesion molecules, cytokines, chemokines, growth factors) have been investigated extensively. In the medicolegal context, the effect of putrefaction on mRNA and proteins of interest is an important consideration. Several studies showed that the level of arginino-succinate lyase mRNA is stable for 18 h postmortem, that of the sodium-coupled neutral amino acid transporter (SNAT2) mRNA is stable for 48 h postmortem, and those of microtubule-associated protein 1A/1B-light chain 3 (LC3)-II and sequestosome 1 (p62) protein are stable for 4 days postmortem [28,46,47]. In contrast, cannabinoid receptor type-2 mRNA was degraded significantly at 3 h postmortem, and matrix metalloproteinase-2 and the tissue inhibitors of metalloproteinase-2 mRNA were significantly degraded at 12 h postmortem [27,48]. The degradation of RNA and protein caused by postmortem effects — especially putrefaction, decomposition, and desiccation — is inevitable after death. Therefore, postmortem changes should be taken into account when selecting markers (i.e. those whose levels remain stable for some time after death). In addition, a control group is required to prevent the confounding effects of the postmortem interval. Seasonal differences in environmental factors should also be considered.

Wound healing, a complex process, is influenced by external and internal factors. Therefore, no single parameter is sufficient for estimating wound age. Use of a combination of parameters can reduce error [49]. Although recent research has focused on the relative expression levels of multiple markers, their baseline expression levels, which differ from their relative levels, have been neglected [50–52]. Biomarker expression levels should be normalized to those of a control group. Gene ontology and pathway analyses allow identification of differentially expressed genes, and genes whose products function in the same pathway tend to have similar patterns of expression. Thus, multiple markers with different expression patterns may be needed to evaluate the timing of an injury accurately.

Furthermore, any factor is detectable in only a proportion of cases at any given time point after wounding [5]. Thus, the ideal marker shows minimal intra-group variability or high homogeneity. Zhu et al. [53] reported that assaying the mRNA levels of multiple reference genes was essential for obtaining accurate data and reducing intra-group variability. They also speculated that the adenylate/uridylate-rich element in the 3’-untranslated region is related to mRNA stability, and mRNA without the adenylate/uri-dylate-rich element exhibits low inter-individual sequence variability. Therefore, the structure and function of markers are important when determining marker homogeneity.

Metabolite levels are direct, accurate indicators of the pathophysiological state of an organism. Metabolo-mics, which involves assaying all low-molecular-weight biochemicals, is used for diagnosing disease, investigating pathogenic mechanisms, and determining prognoses. Metabolic profiling is useful for estimating the postmortem time interval [54,55], but its suitability for determining wound age is unclear. Therefore, assessing changes in factors at various levels of wound healing could enable identification of the biomarkers that would allow us to determine the time of the injury.

The frequency of forensic autopsies of diabetic individuals is increasing. Ji et al. [56] investigated the expression level of receptors for advanced glycation end products during the healing of diabetic wounds in mice. Their results showed that the process of repairing diabetic wounds differs from that of normal wounds, suggesting that the parameters used to assess the age of the normal wound may not be applicable to the diabetic wound. In addition to impaired wound healing in diabetics, some studies showed that healing in patients with peripheral vascular disease is difficult [57–59] and delayed in those with immunosuppression [60]. As things stand, it is necessary to explore other parameters to determine the age of wounds in those with various disease states (compared with the healthy state) that affect wound healing.

Studies involving skin and skeletal muscle samples performed after 2010 are summarized in Table 1. It is obvious from Table 1 that biomarkers were more frequently explored at the morphological and genetic levels than at the protein level. Furthermore, positive histological reactivity of biomarkers was generally observed after 24 h, whereas the changes in mRNA and protein were commonly detected 12 h after injury, relying on the high sensitivity of the methods used. It seems that assays based on mRNA and protein are suitable for estimating the age of early-stage wounds, whereas histology is widely considered to be a reliable method for evaluating later-stage wounds.

**Table 1.**
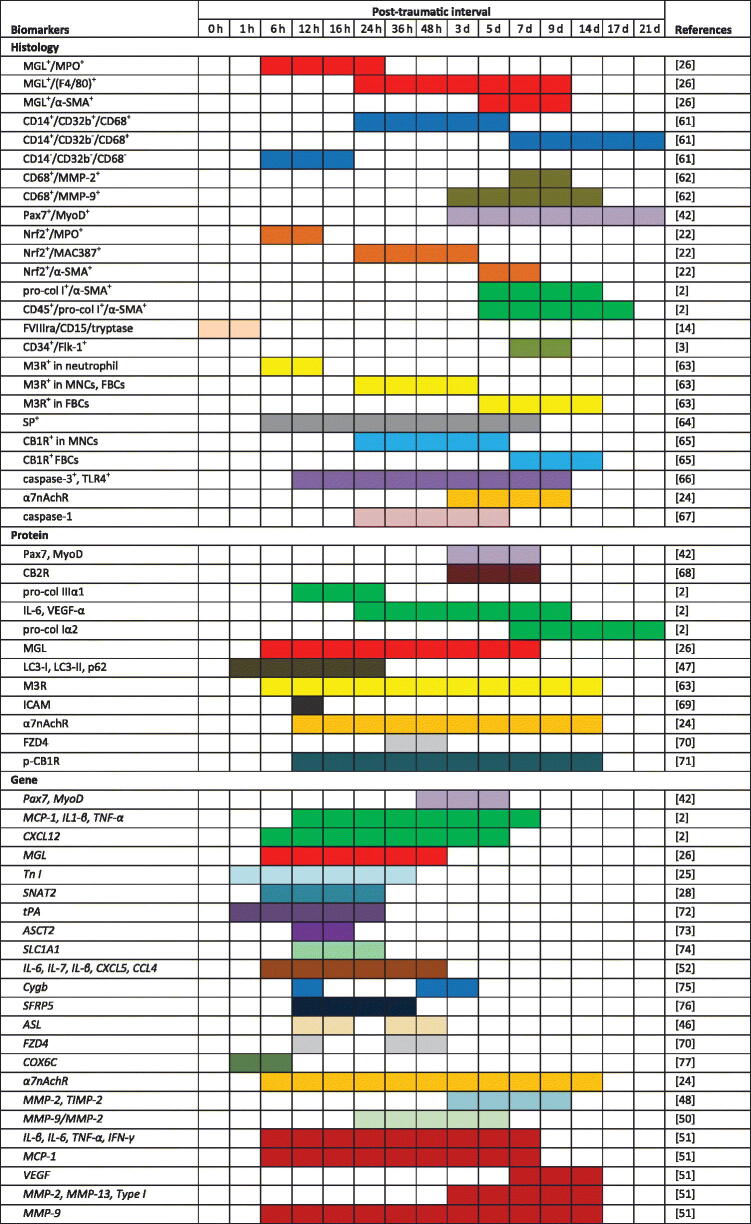
Reactivity of age biomarkers in relation to the time after skin and skeletal muscle injury. Coloured areas showed significant differences between the control and injured groups, markers in different studies appear in different colours.

#### Brain injury

The central nervous system (CNS) is highly sensitive to the deleterious effects of mechanical, ischaemic, and toxic factors. Damaged nervous tissue releases various substances that may have potential as markers of the time elapsed since an injury [78]. Inflammation of the CNS after trauma is similar to that of damaged skin and skeletal muscle, whereas the local reaction (including migration of glial cells) is specific to the CNS [5]. Brain damage is irreversible as neurons are not renewed.

Mechanical brain injury is frequently associated with intracranial haemorrhage, including epidural, subdural, subarachnoid, and brain parenchymal bleeding. Haema-toxylin-eosin and immunohistochemical staining are used to evaluate the age of a haemorrhage [79–81].

Diffuse axonal injury of white matter is one of the most severe consequences of traumatic brain injury and is associated with a high mortality rate. Despite the large amount of research into the pathophysiological mechanisms of diffuse axonal injury, early diagnosis is problematic [38,82,83]. The use of β-amyloid precursor, which translocates from the neuronal cell body to the axon periphery via fast-transport mechanisms, can be detected at the injury site if the axon is disrupted. β-Amyloid precursor is reportedly a specific, highly sensitive marker of axonal damage [84–86].

At the time of the injury, it is common to observe leakage of inflammatory blood cells from damaged tissue and microglial activation following mechanical stimulation. In addition, the robust growth and propagation of reactive astrocytes suggest that they have important roles in wound healing [87]. The dynamics of gliocytes and inflammatory cells (i.e. the substances they release) have been commonly employed to date brain wounds. In 2007, Takamiya et al. [11] suggested that the time-dependent expression of 27 cytokines in cerebral wounds could help estimate wound age. Since 2010, more biomarkers’ expression levels have been surveyed by diverse techniques for wound dating. Markers used to estimate the age of wounds in the brain are shown in Table 2.

**Table 2.** Concentrations of biomarkers with dependence on the survival time after brain injury.

Biomarkers	Technique	Post-traumatic interval									References
		0 h	1 h	6 h	12 h	1 d	3d	5 d	7 d	14 d	
PSD95	WB			+	++	+	+	++	+		[88]
MAP-2	RT-qPCR		+	+		+	+	+	+	++	[89]
β-APP	IHC, WB		+	+	++	+	+	+	+		[90]
	RT-PCR		+	+							[91]
	IHC			+	+	+					[91]
	IHC				+	++	+				[92]
TGF-β1	IHC			+	+	+	++	+	+		[93]
HO-I	IHC			+	++	++	+	+	+	+	[94]
HIF-1α	IHC		++	+	+	+	+	+	+		[95]
CD11b	IHC		+	+	++	+	+	+	+		[96]
IL-6	IHC		+	+	++	+	+				[96]
Caspase-9	ELISA				++	++	+	+	+		[97]
TNF-α	IHC		+	++	+	+	++	+	+		[98]
MMP-9	IHC		+	+	+	+	+	++	+	+	[99]
NTE	IHC				+	++	+	+	+	+	[100]
COX-2	IHC		+	+	+	++	+	+	+		[100]
CaMK-II	IHC, WB				+	+	++	+	+	+	[101]
HAX-1	WB			++	+	+					[102]
Caspase-3	WB			+	+	+	++	+	+		[102]
RAGE	WB, IHC			+	+	++	+	+			[103]
HMGBI	WB			++	+	+					[103]
	IHC		+	++	+	+					[103]
c-Fos	IHC		+	+	+	+	++	+	+		[104]
c-Jun	IHC		+	++	+	+	+	+	+		[104]
vWF	IHC				+	++					[92]
NFL	IHF				+	++	+				[92]
IL8	IHC						+	++	+	+	[105]

IHC: immunohistochemical; IHF: immunofluorescence; WB: Western blotting; +: significant difference between the control and injury groups; ++: the greatest change during the post-trauma period.

### Data analysis and application

The method used to extract useful information from data obtained by diverse techniques for wound-age evaluation is important. Most studies simply approximate the time of injury using the expression patterns of indicators, which can be bimodal or multimodal, resulting in conflicting results for wound age. For this reason, Sun et al. [49] developed an up-regulation/no-change/down-regulation model comprising four mRNAs, which yielded narrower ranges for the age of wounds. Yagi et al. [61] used immunohistochemistry to evaluate cluster of differentiation (CD)—14, CD32B, and CD68 expression in human skin wounds, which exhibited greater specificity and reduced the wound age range compared with the assessment using a single marker. Moreover, for accurate wound-age estimation, van de Goot et al. [106] and Fronczek et al. [8] developed probability scoring systems for the morphological analysis of various indicators. Although these methods yield much information and suggest the utility of wound-age estimation using multiple markers, accurate evaluation of the timing of an injury is hampered by the influence of operator’s skill and the many factors involved in injured tissue repair.

Estimating the time of wounding is thus affected by individual variation, degree of damage, postmortem interval, and the storage conditions of the sample. Mathematical modelling has contributed to analyses of other complex systems (e.g. weather, the economy) and thus may also be applicable to determining wound age in the forensic setting.

### Problems and future perspectives

Wound-age estimation has been a focus of research in recent decades. Determining wound age, particularly at the early stages, largely depends on the experience of the pathologist. The longer one engages in forensic practise, the greater is the knowledge about various factors affecting wound-age estimation, including the age and sex of the deceased, cause of death, and the severity of the injury. Even the most experienced forensic pathologist, however, would welcome the development of an animal model with wounds that takes into account the age of the deceased, the extent of the damage, the age of the wound, the postmortem interval, the different seasons with their environmental changes, and the storage conditions. Because information on autopsy samples is frequently absent or insufficient, animal models, standardized and controlled conditions, and information from dermal samples are needed to obtain reliable results.

Use of multiple markers enables more accurate and reliable determination of wound age. Developments in techniques, particularly high-throughput analysis, have enabled simultaneous analysis of multiple mRNAs and proteins in a single sample. Thus, numerous markers of wound healing have been investigated. Additionally, a method by which to screen for suitable markers is required. Combinations of morphological and molecular techniques — including genomics, proteomics, and metabolomics — will likely to be required to reach objective conclusions.

The extent to which results are applicable to humans and useful for estimating wound age should be assessed. Mathematical modelling has provided guidance for such complex problems as weather forecasting, although it is unclear how their influencing factors could interact with those required for wound dating. Metcalf et al. [107] developed a mathematical model for evaluating the postmortem time interval and obtained promising results. Because wound-age estimation is affected by diverse factors, any mathematical model should be based on data from large-scale animal studies, using the results from human autopsy samples for calibration.

## Conclusion

It is clear that progress in wound-age estimation has been made during the last few years. As technology has advanced at a breathless pace, data access has become easier, and many time-dependent parameters have explored. Although combinations of multiple markers have received considerable critical attention, no system or model has yet been proposed to use such markers for wound aging. The challenge now is how to analyse and utilize the data that have been obtained and put the results into practise.
